# Risk factors for locoregional recurrence in patients with resected N1 non-small cell lung cancer: a retrospective study to identify patterns of failure and implications for adjuvant radiotherapy

**DOI:** 10.1186/1748-717X-8-286

**Published:** 2013-12-09

**Authors:** Chengcheng Fan, Shugeng Gao, Zhouguang Hui, Jun Liang, Jima Lv, Xiaozhen Wang, Jie He, Luhua Wang

**Affiliations:** 1 Department of Radiation Oncology, Cancer Hospital and Institute, Chinese Academy of Medical Sciences and Peking Union Medical College, Pan jia yuan nan li 17#, Chao Yang District, Beijing 10021, China; 2 Department of Thoracic Surgery, Cancer Hospital and Institute, Chinese Academy of Medical Sciences and Peking Union Medical College, Pan jia yuan nan li 17#, Chao Yang District, Beijing 10021, China

**Keywords:** Non–small cell lung cancer, Locoregional recurrence, Survival, N1 stage, Postoperative radiotherapy

## Abstract

**Background:**

Meta-analysis of randomized trials has shown that postoperative radiotherapy (PORT) had a detrimental effect on overall survival (OS) in patients with resected N1 non–small cell lung cancer (NSCLC). Conversely, the locoregional recurrence (LR) rate is reported to be high without adjuvant PORT in these patients. We have evaluated the pattern of failure, actuarial risk and risk factors for LR in order to identify the subset of N1 NSCLC patients with the highest risk of LR. These patients could potentially benefit from PORT.

**Methods:**

We conducted a retrospective study on 199 patients with pathologically confirmed T1–3N1M0 NSCLC who underwent surgery. None of the patients had positive surgical margins or received preoperative therapy or PORT. The median follow-up was 53.8 months. Complete mediastinal lymph node (MLN) dissection and examination was defined as ≥3 dissected and examined MLN stations; incomplete MLN dissection or examination (IMD) was defined as <3 dissected or examined MLN stations. The primary end point of this study was freedom from LR (FFLR). Differences between patient groups were compared and risk factors for LR were identified by univariate and multivariate analyses.

**Results:**

LR was identified in 41 (20.6%) patients, distant metastasis (DM) was identified in 79 (39.7%) patients and concurrent LR and DM was identified in 25 (12.6%) patients. The 3- and 5-year OS rates in patients with resected N1 NSCLC were 78.4% and 65.6%, respectively. The corresponding FFLR rates were 80.8% and 77.3%, respectively. Univariate analyses identified that nonsmokers, ≤23 dissected lymph nodes, visceral pleural invasion and lymph node ratio >10% were significantly associated with lower FFLR rates (*P* < 0.05). Multivariate analyses further confirmed positive lymph nodes at station 10 and IMD as risk factors for LR (*P* < 0.05). The 5-year LR rate was highest in patients with both these risk factors (48%).

**Conclusions:**

The incidence of LR in patients with surgically resected T1–3N1M0 NSCLC is high. Patients with IMD and positive lymph nodes at station 10 have the highest risk of LR, and may therefore benefit from adjuvant PORT. Further investigations of PORT in this subset of patients are warranted.

## Background

Surgery is the cornerstone of treatment for early-stage non–small cell lung cancer (NSCLC). Due to high recurrence rates following tumor resection, including radical surgery, studies have been carried out on the efficacy of adjuvant therapy. Several randomized trials and meta-analyses have confirmed that platinum-based adjuvant chemotherapy can improve overall survival (OS) in patients with stage II and IIIA NSCLC [1-4]; however, the role of postoperative radiotherapy (PORT) remains controversial. Although studies have found that PORT can reduce locoregional recurrence (LR), no survival benefit was observed. Moreover, meta-analyses of randomized trials and retrospective studies have reported that PORT had a detrimental effect on OS in patients with N0 and N1 NSCLC, despite being beneficial in patients with N2 disease [5-8]. As a consequence, PORT is often recommended for patients with resected pathological N2 disease, but not for those with N0 or N1 disease. However, these studies may have been compromised by limitations such as flaws in the study design and outdated radiation techniques.

The reported crude risk of LR for resected N1 disease is high [9-11], which suggests that a subset of N1 patients with high risk of LR may benefit from further local treatment after surgery. The purpose of the present study was to assess the clinical and pathological risk factors that influence LR in patients with resected N1 NSCLC in order to identify and select those patients at high risk of LR for further studies on PORT.

## Methods

### Study population

This retrospective medical record review study was approved by our institutional review board. The inclusion criteria were as follows: (1) pathologically confirmed T1–3N1M0 NSCLC, (2) consecutive treatment between January 2007 and December 2008 in our hospital, (3) anatomical pulmonary resection with lobecotomy, sleeve lobectomy, bilobecotomy or pneumonectomy and (4) complete records of clinical and pathological data. The exclusion criteria were as follows: (1) positive surgical margins, (2) prior neoadjuvant therapy involving chemotherapy, radiotherapy or PORT, (3) synchronous primary lung tumors or a history of lung cancer and (4) perioperative death. Patients were staged according to the 7^th^ edition of the American Joint Committee on Cancer (AJCC) tumor-node-metastasis (TNM) classification system [12].

### Definition of recurrence

The primary end point of this study was freedom from LR (FFLR). Local recurrence was defined as the recurrence of a tumor at the bronchial stump or anastomosis, and regional recurrence was defined as recurrence in the mediastinum, hilum or supraclavicular fossa. Other sites of recurrence, including contralateral lung and metastatic lymph nodes in the neck or axilla were defined as distant metastasis (DM). Concurrent recurrence was defined as LR and DM being detected within 30 days of each other; otherwise, it was considered sequential recurrence.

The diagnosis of disease recurrence and recurrence sites was based on a review of the medical records, including operative notes, follow-up imaging reports and cytological and pathological reports. All local recurrences were confirmed by sputum cytology or bronchoscopic biopsy. Nodal recurrences were confirmed by biopsy using ultrasound-guided fine-needle aspiration (FNA) or endobronchial ultrasound-guided transbronchial needle aspiration (EBUS-TBNA). New or enlarging lymph nodes (≥1 cm short axis) were confirmed by computed tomography (CT) or through hypermetabolism on positron-emission tomography (PET)-CT, which was found to be consistent with disease progression during subsequent clinical follow-ups. Sites of recurrent lymph nodes were identified using the lymph node map proposed by the International Association for the Study of Lung Cancer (IASLC) [13].

### Follow-up

The median follow-up time was 53.8 months (range, 1.4–81.8 months). Physical examination, chest x-rays and/or CT scans and abdominal ultrasonography were performed every 3 months for the first 2 years following adjuvant chemotherapy, then every 6 months for 5 years and every year thereafter. PET-CT was performed if recurrent disease could not be identified or suspected by routine CT. Other tests were carried out at the discretion of the treating physician.

Of the 199 patients that were eligible for this study, 158 (79.4%) had received adjuvant platinum-based chemotherapy after surgery.

OS was measured from the date of surgery to the date of death from any cause; disease-free survival (DFS) was measured from the date of surgery to the date of LR, DM or death from any cause; FFLR was measured from the date of surgery to the date of identification of LR; freedom from DM (FFDM) was measured from the date of surgery to the date of identification of DM.

### Statistical analysis

The Kaplan-Meier method was used to estimate OS, DFS, FFLR and FFDM. Differences between groups were calculated using the log-rank test. Receiver operating characteristic (ROC) curves were constructed, and the Youden Index was used to determine the optimal cut-off points for part of continuous variables, suh as number of dissected lymph nodes and lymph node ratio. Univariate and multivariate Cox regression analyses were performed to assess risk factors associated with LR. A *P*-value <0.05 was considered statistically significant.

## Results

### Patient characteristics

Patient characteristics and treatments are given in Table 1. The median number of dissected lymph nodes was 21 (range, 3–66). The median number of metastatic lymph nodes was 2 (range, 1–8). The number of dissected mediastinal lymph node (MLN) stations was evaluated from both surgical records and pathological reports; however, some of the results were inconsistent: 78 patients had <3 MLNs recorded in both reports; 86 patients had ≥3 MLNs recorded in both reports; and 35 patients had ≥2 dissected MLN stations recorded in their surgical records but <3 in their pathological reports.

**Table 1:** Results of univariate and multivariate analyses of risk factors for freedom from locoregional recurrence in patients with resected N1 non–small cell lung cancer

**Patient characteristics and treatments**	**No. of patients**	**Univariate analysis FFLR (%)**				**Multivariate analysis**	
		**3-yr**	**5-yr**	**P**	**χ**^**2**^	**P**	**HR (95% CI)**
Sex							
Male	164	81.2	78.1	0.934	0.007		
Female	35	78	72.4				
Age (yrs)							
<65	144	79	78.2	0.942	0.005		
≥65	55	85.6	74.7				
Smoking history							
No	143	69	66.6	0.026	4.958	0.388	0.735 (0.365–1.479)
Yes	56	85.3	81.5				
Diabetes							
No	184	80.8	77	0.787	0.073		
Yes	15	80	80				
Tumor type							
Peripheral	89	78.5	74.1	0.267	1.233		
Central	110	82.4	79.9				
Location							
Upper	114	78.5	77.4	0.868	0.028		
Middle/lower lobe	85	83.8	77.3				
Laterality							
Left	103	81	81	0.243	1.363		
Right	96	80.5	73.7				
Surgical procedure							
Pneumonectomy	54	83.1	80.3	0.662	0.191		
Lobectomy	145	79.9	76.2				
Histology							
Squamous cell	101	81.8	80.5	0.201	1.633		
Non-squamous cell	98	79.3	73.2				
Tumor differentiation							
Well/Moderate	156	80.1	75.3	0.497	0.462		
Poor	43	81.6	73.9				
Pathological stage							
T1	63	81.4	77.5	0.935	0.135		
T2	92	79.9	78.5				
T3	44	81.4	74.2				
Tumor size							
≤5 cm	147	82.6	79.9	0.214	1.545		
>5 cm	52	75.3	70				
Visceral pleural invasion							
No	82	88	86.3	0.025	5.025	0.075	1.882 (0.937–3.778)
Yes	117	75.7	71				
LVI							
No	145	83.6	79.9	0.088	2.905	0.213	1.506 (0.79–2.869)
Yes	54	72.9	69.6				
Bronchoscopy							
Negative/not done	88	77.3	72.7	0.080	3.063	0.721	0.873 (0.415–1.836)
Positive	111	83.5	81.1				
Adjuvant chemotherapy							
No	41	86.1	75.3	0.975	0.001		
Yes	158	79.5	77.9				
No. of dissected LNs							
≤23	121	74.4	71.1	0.007	7.981	0.14	0.539 (0.238–1.224)
>23	78	91.1	87.6				
No. of metastatic LNs							
1	88	83.5	82	0.182	1.785		
≥2	111	78.7	73. 7				
LNR							
≤10%	119	86.6	84.1	0.027	4.871	0.976	1.011 (0.506–2.017)
>10%	80	73.8	69.1				
Dissection of MLN							
IMD	113	73.2	68.5	0.001	11.58	0.003	0.309 (0.142–0.671)
CMD	86	90.7	89		1		
No. of metastatic LN stations							
1	138	81.7	79.9	0.154	2.032		
≥2	61	78.4	70.5				
Positive LNs at station 10							
Yes	54	68.5	65.9	0.01	6.724	0.046	0.527 (0.281–0.989)
No	145	85.3	81.7				

FFLR, freedom from locoregional recurrence; HR, hazard ratio; CI, confidence interval; LVI, lymphovascular invasion; LN, lymph node; LNR, lymph node ratio; MLN, mediastinal lymph node; CMD, complete MLN dissection and examination; IMD, incomplete MLN node dissection or examination.

**Figure 1:**
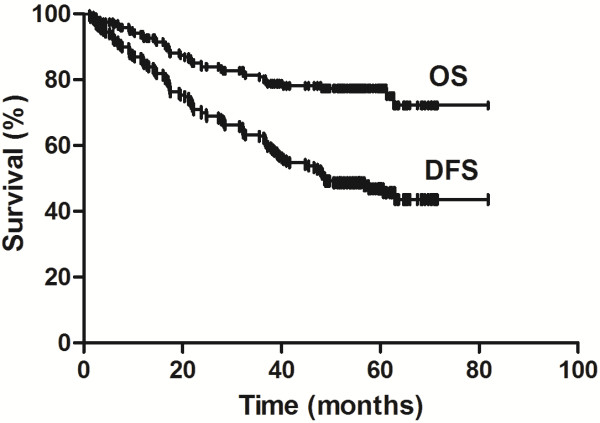
**Survival rates of patients with resected N1 non–small cell lung cancer.** The Kaplan-Meier curves show the decline in overall survival (OS) and disease-free survival (DFS) rates following surgery. The follow-up periods were 1.4–81.8 months (median, 53.8 months).

**Figure 2:**
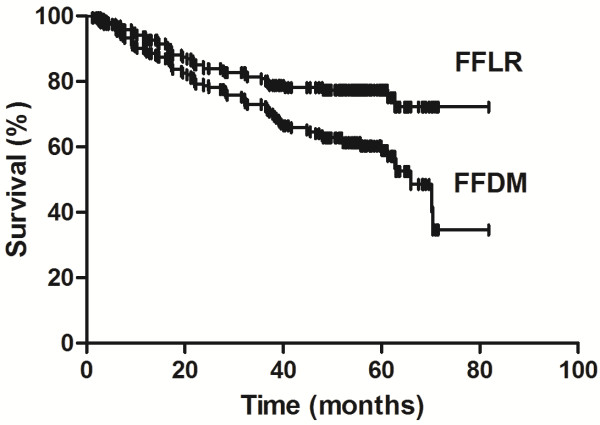
**Freedom from locoregional recurrence and freedom from distant metastasis rates.** The Kaplan-Meier curves show freedom from locoregional recurrence (FFLR) and freedom from distant metastasis (FFDM) rates for patients with resected N1 non–small cell lung cancer following surgery.

The 3- and 5-year OS rates were 78.4% and 65.6%, respectively, and the corresponding DFS rates were 55.3% and 45.7%, respectively (Figure 1). The 3-year and 5-year FFLR and FFDM rates were 80.8% and 77.3%, respectively and 65.9% and 57.4%, respectively (Figure 2). Tumor recurrence occurred in 95 patients (47.7%) by the last follow-up.

**Figure 3:**
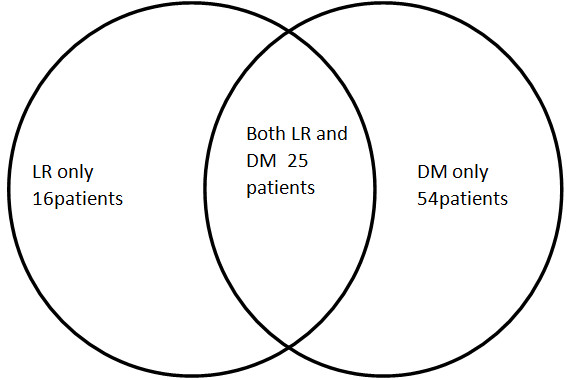
**Pattern of locoregional recurrence and distant metastasis.** The Venn diagram reflects the pattern of locoregional recurrence (LR; left), distant metastasis (DM; right) and concurrent LR and DM (intersection) among the patients in the study group with resected N1 non–small cell lung cancer.

LR and DM were identified in 41 patients (20.6%) and 79 patients (39.7%), respectively (Figure 3). Concurrent LR and DM were identified in 25 patients (12.6%). Lung and pleura were the most common sites of DM (40 patients and 11 patients, respectively). Other DM sites were the brain (24 patients, 12.1%), bone (25 patients, 12.6%), liver (9 patients, 4.5%) and adrenal gland (4 patients, 2.0%).

### Risk factors for LR

We evaluated patients’ clinical and pathological characteristics by univariate and multivariate analyses to identify factors that might be associated with FFLR (Table 1). The optimal cut-off points for the number of dissected lymph nodes and lymph node ratio (LNR) in relation to LR were determined by constructing ROC curves and calculating the maximal Youden index. Complete MLN dissection and examination (CMD) and incomplete MLN dissection or examination (IMD) were defined according to the number of MLN stations recorded in the surgical records and pathological reports. CMD was defined as ≥3 MLNs in both reports with a 5-year FFLR of 89%; IMD was defined as either <3 MLNs in both reports with a 5-year FFLR of 68.6% or as ≥3 MLNs in the surgical records and <3 MLNs in the pathological reports with a 5-year FFLR of 68%.

**Figure 4:**
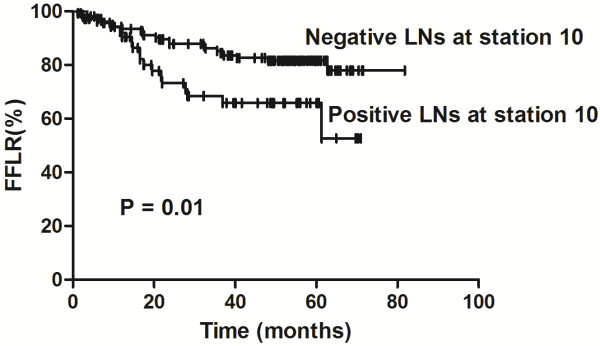
**Comparison between patients with positive and negative lymph nodes at station 10.** The difference in freedom from locoregional recurrence (FFLR) rates between patients with resected N1 non–small cell lung cancer and positive or negative lymph nodes (LNs) at station 10 is significant (*P* = 0.01), showing that a positive LN at station 10 is a risk factor for LR.

**Figure 5:**
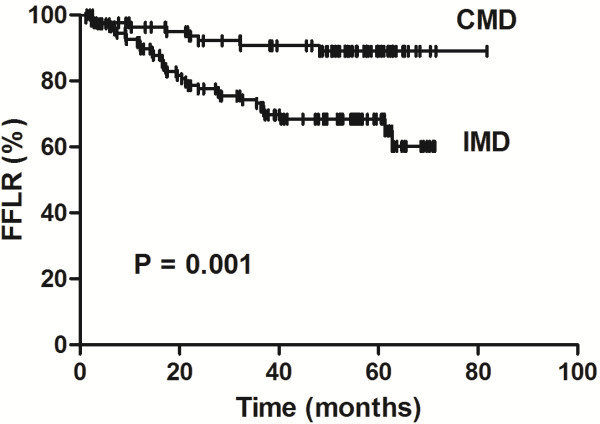
**Comparison between patients with incomplete and complete mediastinal lymph node dissection and examination.** The difference in freedom from locoregional recurrence (FFLR) rates between patients with complete mediastinal lymph node (MLN) dissection and examination (CMD) and incomplete MLN dissection or examination (IMD) is significant (*P* = 0.01), and shows that IMD is a greater risk factor for LR than positive lymph nodes at station 10 in patients with resected N1 non–small cell lung cancer.

Univariate analyses identified the following factors as being associated with a lower FFLR rate: nonsmokers (*P* = 0.026), ≤23 dissected lymph nodes (*P* = 0.007), visceral pleural invasion (*P* = 0.025), LNR >10% (*P* = 0.027); positive lymph nodes at station 10 (*P* = 0.01; Figure 4) and IMD (*P* = 0.001; Figure 5). Other risk factors that showed a trend of lower FFLR included lymphovascular invasion (*P* = 0.088) and negative bronchoscopy findings (*P* = 0.08).

Multivariate analyses showed that positive lymph nodes at station 10 (hazard ratio [HR], 0.527; 95% confidence interval [CI], 0.281–0.989; *P* = 0.046) and IMD (HR, 0.309; 95% CI, 0.142–0.671; *P* =0.003) were associated with a lower FFLR.

**Figure 6:**
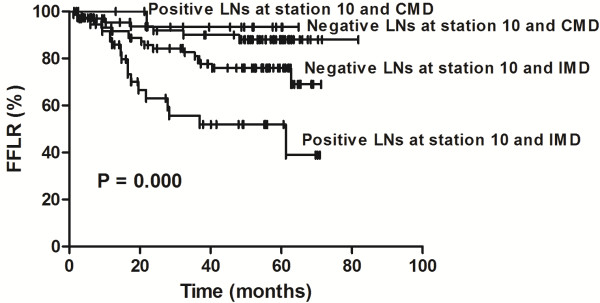
**Comparison of freedom from locoregional recurrence in patients with different risk factors.** Comparison of freedom from locoregional recurrence (FFLR) in patients with resected N1 non–small cell lung cancer and either complete mediastinal lymph node (MLN) dissection and examination (CMD) or incomplete MLN dissection or examination (IMD), and negative lymph nodes (LNs) at station 10 or positive LNs at station 10, shows that those most at risk for LR have both IMD and positive LNs at station 10.

A combined model was generated to evaluate the LR in patents at different risk levels, based on the number of identified risk factors. The 3- and 5-year FFLR rates were as follows: in patients with negative lymph nodes at station 10 and CMD (n = 69), the rates were 90.1% and 88.1%, respectively; in patients with positive lymph nodes at station 10 and CMD (n = 17), the rates were 93.3% and 93.3%, respectively; in patients with negative lymph nodes at station 10 and IMD (n = 76), the rates were 81% and 75.9%, respectively; and in patients with positive lymph nodes at station 10 and IMD (n = 37), the rates were considerably lower at 55.7% and 52%, respectively. The differences in FFLR between patients with one, both or neither of these risk factors were statistically significant (*P* = 0.000; Figure 6).

## Discussion

The LR rate in patients with resected N1 NSCLC is reported to be 9%–46% (Table 2). In comparison, our data showed a 5-year LR rate of 22.7%. LR risk may now be higher as adjuvant chemotherapies can reduce or delay DM and thereby increase survival times. In addition, better LR control has been associated with improved OS in locally advanced NSCLC patients [14,15], indicating that a decreased LR rate after surgery might also improve OS, which suggests that PORT could be beneficial in patients with resected N1 NSCLC who are at high risk for LR.

**Table 2:** Risk factors for locoregional recurrence (LR) and distant metastasis (DM) in patients with resected N1 non–small cell lung cancer (NSCLC)

**Author**	**n**	**CT (%)**	**5-yr actuarial DM (%)**	**5-yr actuarial LR (%)**	**Risk factors for LR**
Saynak [34]	335	8	23 (crude)	35	LVI; tumor size
Higgins [36]	198	25	55	40	Visceral pleural invasion; VATS approach; no. of involved N1 nodes
Varlotto [38]	60	40	32	46	Chemotherapy
Sawyer [30]	107	0	47	38	Positive preoperative bronchoscopy findings
Lopez Guerra [33]	1402	9	16	9	Surgical procedure; LVI; visceral pleural invasion; tumor size
Fujimoto [37]	180	NM	23 (crude)	22 (crude)	Visceral pleural invasion
This study	199	79.4	42.6	22.7	Incomplete MLN dissection; positive LN at station 10

LR, locoregional recurrence; DM, distant metastasis; LVI, lymphovascular invasion; VATS, video-assisted thoracic surgery; LN, lymph node; MLN, mediastinal lymph node; NM, not mentioned.

Comparisons between LR and DM rates for previously reported risk factors and those reported in our study.

To date, studies on PORT have found no survival benefits for patients with resected N1 NSCLC, and therefore, PORT is not recommended for these patients. Moreover, compared with chemotherapy alone, PORT following adjuvant chemotherapy was found to be associated with detrimental survival in N1 disease patients in the Adjuvant Navelbine International Trialist Association (ANITA) randomized study [7]. The ANITA trial was a randomized study, but the patients that were recommended for PORT were selected by the researchers, so were not randomized [7]; consequently, patient selection bias may have been introduced into the results.

A meta-analysis has also reported similar detrimental effects of PORT in N1 NSCLC patients [8]; however, due to improved technologies, PORT may not be detrimental in all patients with N1 disease. The trials included in the meta-analysis had been carried out using older radiation techniques, such as those that delivered doses using wedges with opposed lateral portals with an excessive volume; used different fractionation schedules ranging from 1.8–3.0 Gy; used different total doses ranging from 30–60 Gy; or used cobalt-60, which has a worse biological efficacy and therapeutic ratio than those achieved with current linear accelerators [16-19]. Treatment-related morbidities and deaths were more prevalent with these outdated radiation techniques than with more modern techniques, which may account for the detrimental survival rates. This has been supported by two recent randomized trials on NSCLC, which investigated PORT with modern radiation techniques. The first showed that PORT was associated with lower LR and higher 5-year OS in patients with postoperative pathological N0 NSCLC [20]; the other found that PORT with modern techniques significantly reduced LR, although there no significant improvement in OS and DFS was observed in patients with postoperative pathological stage T1–3 N0–2 M0 NSCLC [21]. The toxicity of PORT was acceptable in both these trials. These findings suggest that PORT based on modern technologies may promote therapeutic efficacy and could be beneficial for patients with resected pathological N1 NSCLC who are at high risk for LR.

To our knowledge, our present study is the first to identify and report that IMD and positive lymph nodes at station 10 may be risk factors for LR in patients with resected N1 NSCLC, and as such, these patients may warrant further attention. Patients with positive lymph nodes at station 10 are at a higher risk for micrometastases in MLNs through the lymphatic drainage pathway of NSCLC. Studies have reported that the number of resected lymph nodes and the extent of dissected lymph node stations were associated with survival in patients with resectable NSCLC [22-24]. Several studies have recommended that at least six lymph nodes from three MLN stations should be excised [23,25,26]; however, others recommend resection of at least six lymph nodes from mediastinal stations and station 10 for accurate nodal staging and prediction of prognosis in patients with resected NI NSCLC [27,28]. Taken together, these results suggest that the extent of dissected or examined MLN stations may be significantly associated with LR and verify the importance of performing sufficient MLN dissections or examinations.

In this study, we found that patients with resected NI NSCLC and <3 dissected or examined MLN stations had lower FFLR rates than those with ≥3 dissected and examinated MLN stations. Furthermore, our findings showed that the 5-year FFLR rate in patients with IMD (75.9%) was worse than that observed in patients with positive lymph nodes at station 10 (93.3%), indicating that IMD was the greater risk factor for LR (Figure 6). Patients with both risk factors had the highest 5-year LR rate (48%), which was comparable to reported values in resected N2 NSCLC [29]; these patients should therefore be recommended PORT. In contrast, patients with IMD alone should only be considered for PORT after careful consultation between the surgeon and radiation oncologist.

Tumor size has been shown to be a prognostic factor for survival in patients with NSCLC [30-32]. Saynak et al. and Lopez Guerra et al. reported that tumor size was independently associated with LR in resected N1 NSCLC patients [33,34]. Other reports include three studies which found that lymphovascular invasion was a risk factor for LR in N1 NSCLC patients [33-35]. However, in the study by Lopez Guerra et al. [33], the number of patients with N1 NSCLC was small (16%) compared to the total sample population, and the 5-year LR was much lower (9%) than those reported in previous studies. Several studies have reported visceral pleural invasion as a risk factor for LR [33,36,37], and Varlotto et al. observed an increased risk of LR following adjuvant chemotherapy [38]. Surgical procedures, such as lobectomy or pneumonectomy and positive preoperative bronchoscopy have also been associated with a risk of LR in N1 NSCLC patients [30,35,39]. However, these reports are inconsistent, and their findings will need further confirmation before being used in patient selection for PORT in N1 NSCLC.

Our retrospective study had the following limitations: It was a single-institution study, and therefore may have had patient selection bias. Some of the patients underwent insufficient MLN dissection or examination, and may have been staged with N2 disease if they had undergone sufficient MLN dissection, which could have introduced staging bias. Patients with concurrent LR and DM may not accurately reflect the pattern of failure observed in this study, and LR that developed before or after DM may not have been identified promptly due to occult symptoms or incomplete radiologic examination. Despite these limitations, our findings support further investigations on the role of PORT in patients with resected N1 NSCLC who have the risk factors for LR identified in this study.

## Conclusions

The incidence of LR in patients with surgically resected T1–3N1M0 NSCLC is high. Patients with both IMD and positive lymph nodes at station 10 are at the highest risk for LR, and potentially, the most likely to benefit from adjuvant PORT. Our findings indicate that further investigation of PORT is warranted in this subset of N1 NSCLC patients.

## Competing interests

The authors declare that they have no competing interests.

## Authors’ contributions

CF drafted the manuscript. SG, ZH, LJ, JML participated in data collection, and helped to analyze the data. JH, SG participated in the coordination of the study. LW conceived of the study, participated in its design. All authors made substantial contributions to acquisition and statistical analysis of data, and read and approved the final manuscript.
